# Silicon Nanomaterials for Biosensing and Bioimaging Analysis

**DOI:** 10.3389/fchem.2018.00038

**Published:** 2018-02-28

**Authors:** Xiaoyuan Ji, Houyu Wang, Bin Song, Binbin Chu, Yao He

**Affiliations:** Laboratory of Nanoscale Biochemical Analysis, Institute of Functional Nano and Soft Materials and Jiangsu Key Laboratory for Carbon-Based Functional Materials and Devices, Collaborative Innovation Center of Suzhou Nano Science and Technology, Soochow University, Suzhou, China

**Keywords:** silicon, nanomaterials, synthesis, biosensing, bioimaging

## Abstract

Biochemical analysis in reliable, low-toxicity, and real-time manners are essentially important for exploring and unraveling biological events and related mechanisms. Silicon nanomaterial-based sensors and probes have potentiality to satisfy the above-mentioned requirements. Herein, we present an overview of the recent significant improvement in large-scale and facile synthesis of high-quality silicon nanomaterials and the research progress of biosensing and bioimaging analysis based on silicon nanomaterials. We especially illustrate the advanced applications of silicon nanomaterials in the field of ultrasensitive biomolecular detection and dynamic biological imaging analysis, with a focus on real-time and long-term detection. In the final section of this review, we discuss the major challenges and promising development in this domain.

## Introduction

During the past decades, functional nanomaterials [e.g., fluorescent semiconductor quantum dots (QDs), graphene, carbon nanodots, gold/silver nanoparticles (Au/Ag NPs), etc.] have been intensively employed for the design of various biosensors and probes, owing to their excellent physicochemical properties (e.g., unique optical/electronic performance, large ratios of surface-to-volume, and good surface tailorability as well as abundant surface chemistry, etc.) (Jung et al., 2010; Holzinger et al., 2014; Tilmaciu and Morris, 2015). With the rapid development of silicon nanotechnology, silicon nanostructures/nanohybrids with attractive properties have been extensively developed for the rational fabrication of high-quality sensors and probes for bioimaging and biosensing applications (Nishimura et al., 2013; Wang et al., 2013; Lai et al., 2016). It is worth pointing out that silicon nanomaterials [e.g., silicon nanoparticles (SiNPs), silicon nanoneedles] could easily biodegrade into renal clearable molecules (i.e., silicic acid) and then excrete out the body with no evidence of toxicity *in vivo* (Park et al., 2009; Chiappini et al., 2015). Of particular concern is that ultra-small (diameter: 3–10 nm) Si NPs have received the Food and Drug Administration (FDA)-approved investigational new drug approval for first-in-human clinical trials (Phillips et al., 2014). Consequently, different dimensional silicon nanomaterials have been prepared and functionalized for various analytical applications. For instance, zero-dimensional fluorescent SiNPs featuring good water-dispersibility, strong fluorescence, as well as ultrahigh photostability, have been proved to be ideally suitable for tracking live cells in real-time and long-term ways (Peng et al., 2014; Zhong et al., 2015). On the other side, one-dimensional silicon nanowires (SiNWs) and two-dimensional silicon wafer nanohybrids (e.g., silicon wafer decorated with metal NPs) could be designed as a general biosensing technology for enhanced surface-enhanced Raman scattering (SERS) studies (Wipf et al., 2013; Wang et al., 2016). Compared with free metal NPs-based SERS sensors, silicon-based SERS sensors embody superior sensitivity and reproducibility. Consequently, various kind of functional silicon SERS sensors have been exploited for sensitively and selectively detecting myriad biological and chemical species in reliable and reproducible manners.

**Scheme 1:**
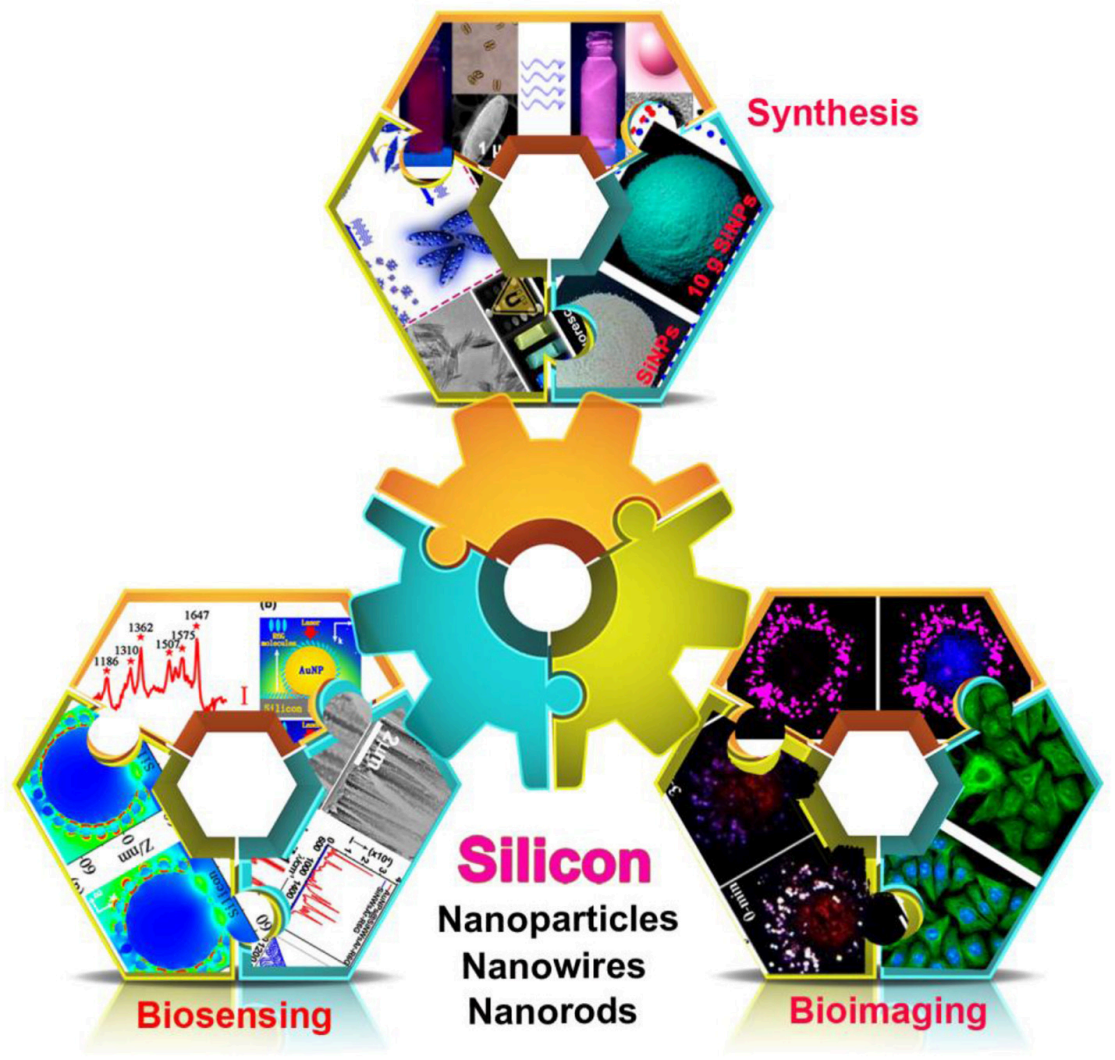
Fabrication of silicon nanomaterial-based platform for biochemical applications [i.e., synthesis of silicon nanomaterials (Wu S. C. et al., 2015) (reprinted with permission, Copyright 2015, ACS Publications); (Zhong et al., 2015) (reprinted with permission, Copyright 2015, ACS Publications); (Song et al., 2016) (reprinted with permission, Copyright 2016, ACS Publications), biosensing (Sun et al., 2015) (reprinted with permission, Copyright 2015, ACS Publications); (Wang et al., 2014) (reprinted with permission, Copyright 2014, AIP publications); (Zhu et al., 2015) (reprinted with permission, Copyright 2015, ACS Publications), and bioimaging (Zhong et al., 2013) (reprinted with permission, Copyright 2013, ACS Publications); (Wu S. C. et al., 2015) (reprinted with permission, Copyright 2015, ACS Publications); (Ji et al., 2015) (reprinted with permission, Copyright 2015, Wiley-VCH)].

Herein, this review article will briefly summarize recent significant improvement in the preparation of silicon nanomaterials and their bioapplications in biochemical analysis (Scheme 1). In the following sections, we first present recent efforts in preparing fluorescent silicon nanomaterials with high luminescence in facile and large-scale manners. Then, we illustrate the typical examples of sensors and bioimaging analysis based on silicon nanomaterials. In the last section, we examine future challenges and potentialities associated with myriad biochemical analysis studies based on silicon nanomaterials.

## Synthesis of silicon nanomaterials

Since the first discovery of the unique optical properties of fluorescence silicon nanomaterials (Wilson et al., 1993; Park et al., 2001), numerous synthetic strategies have been reported to prepare silicon nanomaterials with high fluorescence and photostability, vastly facilitating the exploration of their optical applications in biochemical analysis (Atkins et al., 2011; Zhou et al., 2015; Dasog et al., 2016; Liu et al., 2016). Combined with current research concerns, this section intends to briefly summarize recent progresses in facile and large-quantity synthesis of fluorescence silicon nanomaterials, including zero-dimensional SiNPs and one-dimensional silicon nanostructures.

Various high-efficacy and workable strategies have been introduced for the large-scale preparation of fluorescence SiNPs with strong fluorescence [photoluminescent quantum yield (PLQY): ~15–25%] and robust photostability in facile and rapid manners, which are fundamentally critical for their long-awaited applications. By virtue of fast rise of temperature and uniform heating of samples, microwave irradiation is advantageous for large-scale and rapid synthesis of high-quality fluorescent SiNPs. As a typical example, Zhong et al. reported that 0.1 g SiNPs could be readily obtained within 10 min by using organosilicon molecules as silicon precursors via microwave-assisted method (Zhong et al., 2013). Recently, by using low-cost and non-toxic silicon resources (e.g., rice husks, wheat straws, and diatoms, etc.), a microwave-assisted biomimetic method was further developed for synthesizing SiNPs in an environmentally friendly manner (Wu S. C. et al., 2015; Wu et al., 2016). Besides aforementioned microwave equipment-assisted strategy, a photochemical method has gained researchers' attention, which can be used to prepare SiNPs in glass flasks under mild conditions (i.e., room temperature and normal pressure) (Zhong et al., 2015). Of particular note, ~10 g high-quality SiNPs could be obtained in short time (<40 min) under UV irradiation using a potable xenon lamp, which sufficiently satisfied the need of wide-ranging biological applications.

Different from zero-dimensional SiNPs, one-dimensional fluorescent silicon nanostructures possess unique optical properties (Zheng et al., 2012; Wu K. F. et al., 2015). Particularly, reduced thresholds of multiexciton generation and optical gain of one-dimensional silicon nanostructures are beneficial for the fabrication of high-performance silicon nanomaterial-based nanolasers and nanodevices (Shabaev et al., 2013). The pioneering example of one-dimensional fluorescent silicon nanorods (SiNRs) has been reported in 2013 (Lu et al., 2013). In this work, relative low luminescence (PLQY: ~5%) SiNRs have been prepared by the decomposition of trisilane in hot squalane with the presence of Tin NPs and odecylamin, followed by hydrogen fluoride (HF) and thermal treatment. Recently, on the basis of microwave-assisted synthetic approach for preparing SiNPs (Zhong et al., 2013), highly luminescent SiNRs with PLQY of ~15% have been further fabricated by adding milk into the reaction precursors (Song et al., 2016). Briefly, crystal nucleation including silicon and carbon nanoclusters could be firstly created through microwave irradiation. Meanwhile, calcium phosphate (Cap) crystallization was formed through fusion-fission between Ca or P ions linked protein micelles in the presence of aminosilane, which facilitated the aggregation of the silicon and carbon nuclei, resulting in one-dimensional silicon nanomaterials with rod-structures. Particularly, the as-prepared SiNRs possessed excitation wavelength-dependent fluorescence spectra and have been conceptually developed for the construction of white-light-emitting devices (LEDs). Lately, the same group introduced a new type of one-dimensional multifunctional silicon shuttles (SiNSs), which could be obtained by addition of Fe^3+^ ions into the same silicon source. Following the above-mentioned workflow, SiNSs were fabricated through Fe^3+^-induced oriented attachment mechanism (Song et al., 2017). Significantly, the resultant SiNSs featuring intrinsic magnetism and excitation-wavelength dependant luminescence simultaneously were proved to be superbly suitable for advanced anti-counterfeiting application with additional magnetism-related secrecy (Song et al., 2018). As thus, benefiting from advantages of microwave, the presented strategies have been proved to be efficient and general synthetic approaches for preparing one-dimensional silicon nanomaterials rapidly and facilely; and moreover, such method shows great promise for developing fluorescent silicon nanomaterials with multiple functionalities.

## Biosensing

The past decade has witnessed the exciting achievements in the fields of silicon nanomaterials/nanohybrids-based sensors, enabling determination of myriad biological and chemical species in sensitive and reliable manners. In this section, we focus on introducing typical recent advances of silicon-based biosensors, particularly including field-effect transistor (FET) sensors, fluorescent sensors and surface-enhanced Raman scattering (SERS) sensors.

**Figure 1:**
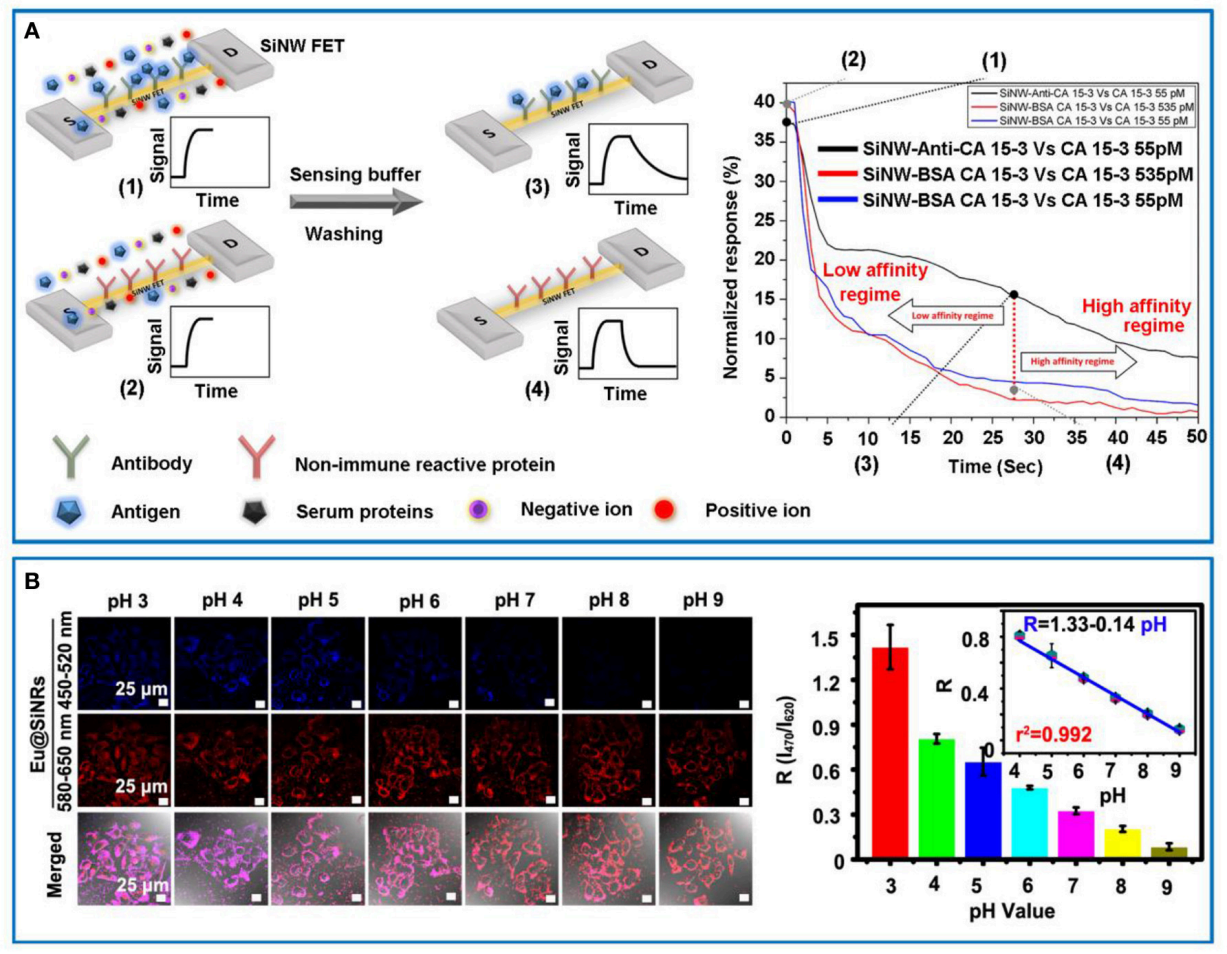
**(A)** Schematically illustrating the workflow of antibody-conjugated SiNW-based FET sensor device (upper part, green receptor units) vs. non-immune reactive protein-conjugated control device (lower part, red receptor units) (left panel). Right panel: Specific (anti-CA 15-3) and non-specific protein (BSA CA-15-3) functionalized SiNW-based FET sensor devices for the detection of the CA 15-3 antigen. Reprinted with permission from Krivitsky et al. (2016). Copyright (2016) ACS Publications. **(B)** Eu@SiNRs for intracellular pH measurement. Confocal images of internalized Eu@SiNRs in MCF-7 cells with different cytoplasmic pH values (i.e., 3–9) (left panel). Scale bars = 25 μm. Right panel: Corresponding histograms of the fluorescence intensity ratio (*R* = *I*_470_ /*I*_620_) vs. pH values ranging from 3 to 9. Inset is the linear relationship between *R* and pH values (i.e., 4–9). Reprinted with permission from Chu et al. (2017). Copyright (2017) ACS Publications.

The FET sensors can evidently amplify electronic signals, which are mainly composed of a semiconductor path (defined as “channel”) and two electrodes (defined as “source” and “drain,” respectively) (Knopfmacher et al., 2014). Specifically, the conductance signals of FET sensors could vary when detecting biological or chemical species, which would induce a negative or positive gate voltage. To date, SiNWs-based FET sensors have been used for real-time, label-free, sensitive, and multiplexed determination of a variety of species, including chemical reagents as well as biological species [e.g., sodium ions (Wipf et al., 2013), nucleic acids (Gao et al., 2013; Lu et al., 2014), cancer biomarker (Shehada et al., 2014), and proteins (Krivitsky et al., 2016) etc.]. Recently, Krivitsky et al. reported a simple and efficient strategy for sensing specific biomarkers directly from unprocessed biosamples using antibody-modified SiNW-based FET devices, which was free of time-consuming manipulation procedures (Krivitsky et al., 2016). As illustrated in Figure 1A (left panel), when the biosample contained analyte biomarker, the specific binding of biomarker on antibody-modified SiNW FET device would result in relative slower rate of returning to baseline during the “dissociation regime” compared to those of control groups (e.g., SiNW modified with non-specific antibody or no antigen in the sample). Based on the presented sensitive and selective approach, the mouse monoclonal antihuman cancer 15-3 IgG (CA 15-3) in practical sample was readily detected and quantified by using the anti-CA 15-3-modified SiNW FET sensing device based on the corresponding dissociation kinetic curves (Figure 1A, right panel). In addition to the conventional affinity-based FET sensors, C. Lieber's group also developed several types of SiNWs-based transistors for recording neural activity in multiplexed, long-term, and high-resolution manners (Qing et al., 2014; Xie et al., 2015). For example, they stereotaxically implanted the 3D mesh-based nanoelectronics incorporated with SiNWs-based FET sensors in a frozen state into rodent brains with minimal damage, and employed it to record multiplexed local field potentials (LFPs) and single-unit action potentials from the somatosensory cortex, opening up new avenues for implantation and long-term brain activity mapping based on silicon nanomaterials (Xie et al., 2015).

Fluorescent sensors feature excellent sensitivity, short-time data acquisition, facile manipulations and low cost, which have been intensively explored for a myriad of sensing applications. By virtue of strong and stable fluorescence of silicon nanomaterials, various types of fluorescent silicon nanomaterial-based sensors have been designed and fabricated for the detection of biological and chemical species, including glucose (Yi et al., 2013a), agricultural chemicals (Yi et al., 2013b), nitroaromatic explosives (Gonzalez et al., 2014; Ban et al., 2015), food additives (Jose et al., 2016), and intracellular pH (Chu et al., 2016, 2017), and so forth. Very recently, Chu et al. presented one dimensional europium (Eu)-doped SiNRs-based ratiometric sensing system without additional chemical modification, allowing for detecting intracellular pH fluctuation in live cells in real-time and long-term manners (Chu et al., 2017). Particularly, the presented Eu@SiNRs featured pH-sensitive emission peak at 470 nm and pH-insensitive one at 620 nm simultaneously under single-wavelength excitation, thus producing ratiometric signals (*R* = *I*_470_/*I*_620_). Remarkably, the developed sensors exhibited broad-pH response (e.g., ~3–9) in human breast cancer (MCF-7) cells (Figure 1B, left panel), which was confirmed by corresponding liner regression equation and correlation coefficient (Figure 1B, right panel). The instinct fluorescence emission change of Eu@SiNRs probe with pH fluctuation eliminated linking of pH-sensitive moiety and further modification of reference fluorophores, providing novel strategies for facile fabrication of high-quality ratiometric sensors based on fluorescent nanomaterials.

SERS is able to amplify the feeble Raman intensity ideally up to 10^14^~10^15^, offering ultrasensitive avenues to explore the Raman signals at the single-molecule level. Compared to free Au NPs or Ag NPs-based SERS-active substrates, silicon nanohybrids (Au/Ag NPs-decorated silicon wafer or SiNWs array)-based SERS substrates feature superior SERS enhancement and better reproducibility (Shi et al., 2017). The distinct SERS enhancement is originated from the hybridization of metal nanoparticles-scattered electromagnetic field and Si-reflected electromagnetic field (Wang et al., 2014). Meanwhile, the improved reproducibility is ascribed to uniform metal nanoparticles tightly anchored on the silicon surface, efficiently avoiding the uncontrollable aggregation of free nanoparticles in liquid phase (Wang et al., 2016). Taking advantages of these merits, silicon nanohybrids-based SERS substrates are ideally suitable for the analysis of myriad biological and chemical samples in practical systems in sensitive, reliable and reproducible manners, such as apoptotic cell (Jiang et al., 2013), mercuric ion (II) (Sun et al., 2015), DNA (Zhu et al., 2015), bacteria (Wang et al., 2015), lead ions (Shi et al., 2016), and trinitrotoluene (TNT) (Chen et al., 2017). On the basis of these exciting works, silicon nanohybrids-based SERS sensors have been well-designed as portable and reliable analytical platforms, which serve as powerful tools for tracing specific compound from environmental samples.

## Biological imaging

SiNPs featuring benign biocompatibility and unique optical properties (i.e., strong fluorescence coupled with ultrahigh photostability) are emerging as novel high-quality fluorescent nanoprobes for biological imaging analysis, particularly for tracking dynamic biological procedures in long-term and real-time manners.

Systematic characterizations of bio-behaviors of SiNPs in biological systems (e.g., cellular internalization mechanism, intracellular trafficking, and final destination, etc.) are crucial for reliable toxicology analysis, providing a feasible evaluation of utilizing SiNPs for biological applications. By virtue of strong and stable fluorescent signals, the cellular behaviors of SiNPs could be dynamically monitored in live cells (Shiohara et al., 2010, 2011; Cao et al., 2017; Zhou et al., 2017). Very recently, comprehensive and reliable investigations of cellular internalization and intracellular fate of SiNPs have been revealed by analyzing the colocalization of SiNPs with various subcellular compartments (Cao et al., 2017). Typically, SiNPs were internalized into cells mainly through clathrin-mediated and caveolae-dependent endocytosis and actively transported from periphery to the perinuclear region along microtubules after cellular internalization. Along with efficient internalization, SiNPs showed no apparent toxic effect on cell growth, as demonstrated by the metabolic activity and integrity of the plasma membrane. Besides above-mentioned cellular investigations *in vitro*, visual observation of *in vivo* behaviors of SiNPs was further achieved, facilitating the extensive utilization of SiNPs for biological and biomedical applications (Zhou et al., 2017). By using *C. elegans* as a classic model organism, biodistribution, stability, and biocompatibility of SiNPs in live organisms have been elucidated. The distribution of SiNPs could be altered by different administration methods; and moreover, the internalized SiNPs would reserve in specific organisms without diffusion during long-term observation time (i.e., 4 h), demonstrating the possibility of using SiNPs-based bioprobes for specific tissue imaging studies. In addition, SiNPs have a little or no toxic effect on body morphology, life span, and reproduction ability of tested worms, implying the superb biocompatibility of SiNPs in living organisms. These findings suggest the possibility for design of high-quality biocompatible SiNPs-based bioprobes for long-term and real-time tracking biological events *in vitro* and *in vivo*.

**Figure 2:**
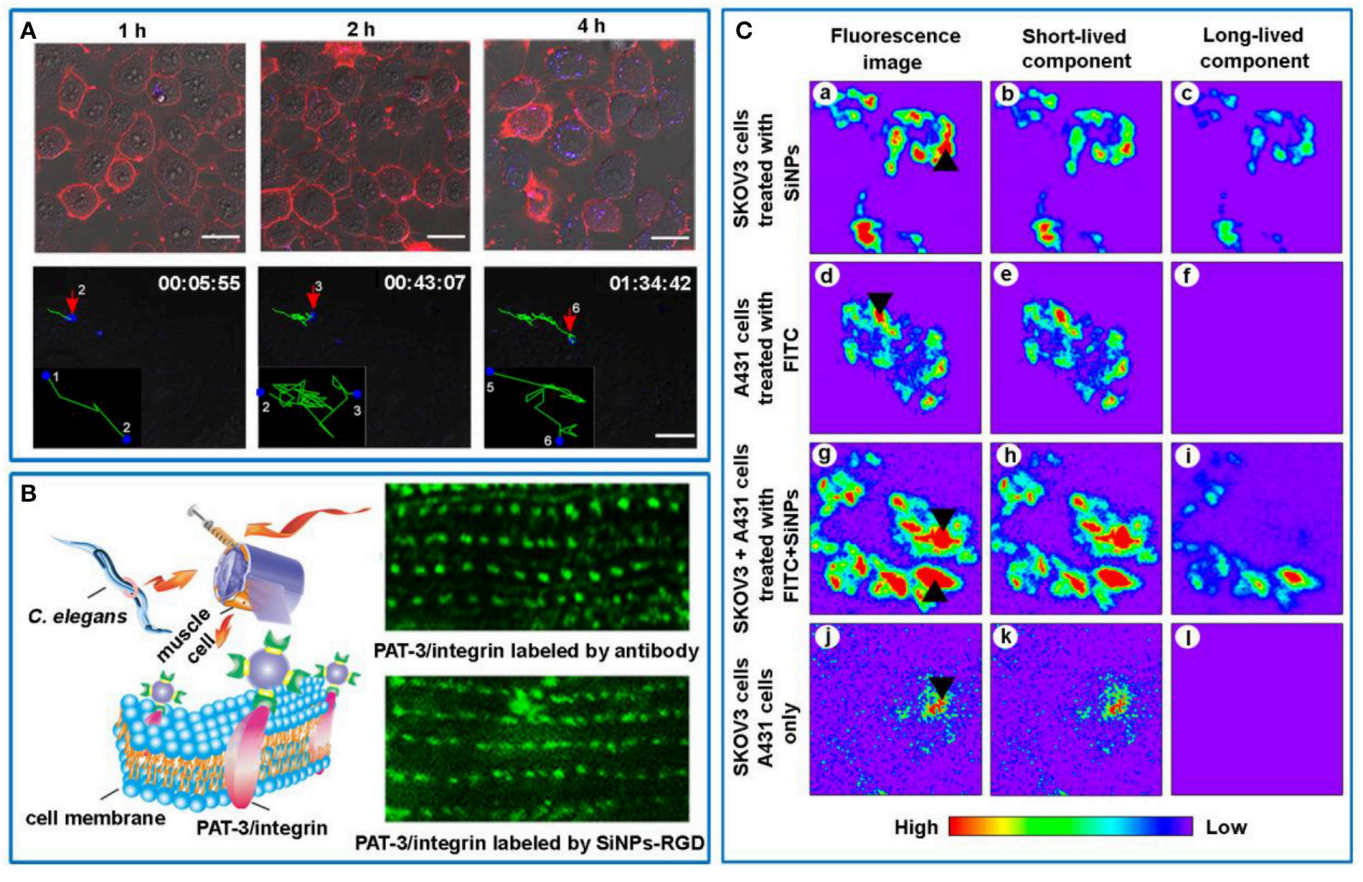
**(A)** Confocal images of time-dependent cellular uptake of PEI-SiNPs/pDNA nanocomplexes in HeLa cells (top panel). Cell membranes were stained with Dil. Fluorescence of SiNPs and Dil is defined as blue and red, respectively. Scale bars = 20 μm. Bottom panel: Real-time and long-term tracking the dynamic movement of the nanocomplexes in a live cell. The movement trajectory is delineated in green line. Reprinted with permission from Pang et al. (2016). Copyright (2016) Springer. **(B)** Schematic illustration of SiNP-RGD for labeling PAT-3/integrin at the muscle cell membrane in *C. elegans* (left panel). Right panel: Specific labeling the subcellular PAT-3/integrin using SiNP-RGD and PAT-3 antibody. Reprinted with permission from Zhou et al. (2017). Copyright (2017) Springer. **(C)** Fluorescence images and time-gated confocal images of SKOV3 cells immunostained by anti-HER2-modified SiNPs **(a–c)**, A431 cells stained by anti-mouse secondary antibodies-labeled FITC **(d–f)**, co-cultured SKOV3 cells and A431 cells labeled by anti-HER2-modified SiNPs and FITC coupled with anti-mouse secondary antibodies, respectively **(g–i)**, and non-treated SKOV3 cells and A431 cells **(j–l)**. Reprinted with permission from Tu et al. (2017). Copyright (2017) ACS Publications.

Biologically relevant molecules [e.g., transferrin (Tf) (Nishimura et al., 2013), sugar (Lai et al., 2016), targeting peptides (Song et al., 2015; Zhou et al., 2017), and polyethylenimine (PEI) (Pang et al., 2016), etc.] have been utilized to functionalize SiNPs, producing the SiNPs-based biofunctional nanoprobes for dynamically studying carbohygrate-carbohydrate interactions, single Tf receptor (TfR) molecule tracking, and targeting specific cancer cells. In particular, benefiting from the non- or low toxicity and high photostability of SiNPs, SiNPs-based fluorescent probes are superbly suitable to track cell-biological interactions in living cells in a real-time way, which have been studied by several groups. In 2016, Pang et al. developed PEI encapsulated SiNPs nanocomposites, which simultaneously possessed bright and stable fluorescence, high DNA-binding capacity (~97%), and adaptable transfection efficiency (~35%) in human cervical carcinoma (HeLa) cells as well as feeble cytotoxicity (Pang et al., 2016). Taking advantages of these merits, the dynamic transport of internalized SiNPs-based carriers could be monitored by detecting stable and bright blue fluorescence signals of SiNPs (Figure 2A, top panel). The entire trajectory presented that SiNPs-based gene carriers moved toward perinuclear region, which was advantageous to efficiently delivery genetic information into nucleus (Figure 2A, bottom panel). As a typical example, by functionalizing SiNPs with cyclic RGD (i.e., arginine-glycine-aspartic acid) peptides, the resultant SiNPs-RGD bioprobes allowed for monitoring integrin-mediated endocytosis during persistent observation time (~120 min) (Song et al., 2015). Such SiNPs-RGD bioprobes were further proved to be suitable for specifically labeling and imaging of body-wall muscle cells in live *C. elegans* by targeting PAT-3/integrin at a molecular-level (Figure 2B) (Zhou et al., 2017). These works suggest that RGD functionalized SiNPs can be used as a general tool for *in vitro* and *in vivo* bioimaging analyses, which is also confirmed by cancer-related *in vivo* applications addressed by Erogbogbo et al. (2011) and Ji et al. (2015) independently.

It is worth noting that the imaging resolution of SiNPs-based fluorescence imaging can be dramatically improved by using time-gating techniques (Gu et al., 2013; Joo et al., 2015; Kim et al., 2017; Tu et al., 2017). Early in 2013, Gu et al. utilized photoluminescent porous SiNPs (pSiNPs) with unusually long-emission lifetime (5–13 μs) for time-gated imaging of tissues *in vivo*, completely eliminating shorter-lived (10 < ns) emission signals from fluorescent proteins or tissue autofluorescence (Gu et al., 2013). In particular, pSiNPs-administrated tumor displayed distinct fluorescence, whereas autofluorescence of normal tissue and short-lived fluorescence of mCherry-expressing tumor were completely removed in the TG image. Later, Tu et al. further demonstrated the fluorescence signals of SiNPs with long photoluminescence lifetimes of ca. 25 μs could be separated with shorted-lived fluorescein isothiocyanate (FITC) by using TG confocal fluorescence imaging regardless of their overlapped photoluminescence spectra (Tu et al., 2017). Typically, as shown in Figure 2C, co-cultured SKOV3 (human ovarian carcinoma cells) and A431 (human epidermoid carcinoma cells) cancer cells immunostained by functionalized SiNPs and FITC could not be separate from each other in fluorescence imaging and short-lived component, whereas only SiNPs-labeled SKOV3 cells exhibited fluorescence signals in long-lived component. These demonstrations imply that long-lived SiNPs-based TG imaging technique has great potential for high-contrast and high-sensitivity optical imaging, such as precise discernment of tumor margins during surgery without disturbing adjacent normal tissues with background autofluorescence or interfering chromophores with short fluorescence lifetimes.

## Conclusion and perspective

In conclusion, past several years have witnessed considerable progresses in the fabrication of silicon nanomaterials and their applications in biochemical analysis. Several economic and facile synthetic strategies have been developed for the preparation of strong fluorescent SiNPs with controllable colors in facile and large-quantity manners. Meanwhile, effective methods of surface modification have been reported to further improve optical properties and aqueous dispersibility of SiNPs. Besides the zero-dimensional fluorescent SiNPs, one-dimensional fluorescent silicon nanostructures (e.g., SiNRs and SiNSs) have been fabricated. Current challenge remains that the exact photoluminescence mechanism of fluorescent silicon nanostructures is controversially to some extent, which requires thorough elucidation in the future.

In terms of sensing applications, benefiting from superior optical properties (i.e., strong and stable fluorescence), SiNPs have been designed as diversified fluorescent sensors, and silicon nanohybrids-based substrates have been employed for the fabrication of high-performance SERS sensors. Such high-quality sensing platform featuring high sensitivity, favorable specificity, and excellent reproducibility, is extremely suitable for the determination and analysis of chemical reagents and biological species in reliable and sensitive manners. Notwithstanding, it is worth pointing out that current sensing applications are mostly limited in the lab research, extensive effort is therefore required to improve the consolidated feasibility of the silicon-based sensors for measurement and analysis of practical samples. On the other hand, silicon nanomaterial-based SERS database featuring superior SERS enhancement and better reproducibility can be collected and served as input data for SERS spectra-based artificial intelligence sensing application, potentially assisting artificial intelligence (AI) to make decisions in sensitive and reliable manners. For bioimaging fields, by the virtue of the superior optical properties (i.e., robust fluorescence coupled with ultrahigh photostability), SiNPs have been employed as novel promising fluorescent nanoprobes, which enable monitoring dynamic biological procedures in long-term and real-time manners. Despite of these exciting research advances on the exploitation of SiNPs for biological imaging analysis, deep investigations are still necessary to explore the potential feasibility for clinic cancer treatment (e.g., intraoperative imaging and surgical excision of sentinel lymph nodes).

**Scheme 2:**
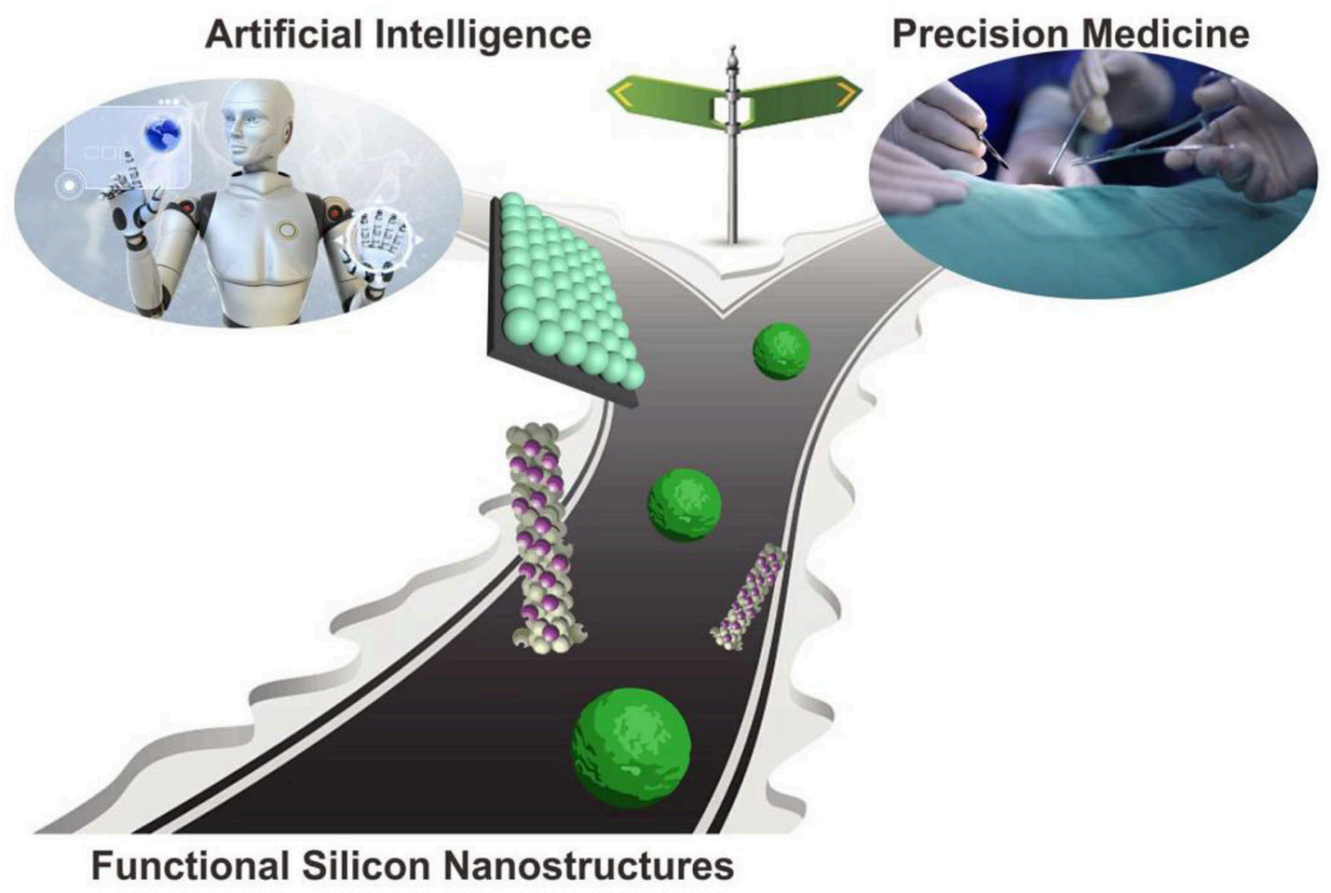
Perspective of silicon nanotechnology in biochemical analysis (Chu et al., 2017, reprinted with permission, Copyright 2017, ACS Publications).

We believe that accompanied by deepening understanding of the above-mentioned challenges, the silicon-based bioimaging and nanosensors would raise new perspectives for various biochemical analysis studies (Scheme 2), and show great potentiality for extensive practical applications in biochemical analytical and sensors fields (e.g., artificial intelligence and precision medicine).

## Author contributions

XJ, HW, BS, and BC reviewed literatures and wrote the manuscript text. YH reviewed the article.

### Conflict of interest statement

The authors declare that the research was conducted in the absence of any commercial or financial relationships that could be construed as a potential conflict of interest.
